# Delirium management in critically ill patients

**Published:** 2013

**Authors:** Enrique Calvo-Ayala, Babar Khan

**Affiliations:** 1 Division of Pulmonary, Allergy, Critical Care, Occupational and Sleep Medicine, Department of Medicine, Indiana University School of Medicine, Indianapolis, IN; 2 Indiana University Center for Aging Research, Indianapolis, IN; 3 Regenstrief Institute, Inc., Indianapolis, IN, USA

**Keywords:** intensive care unit, critical illness, acute brain dysfunction, delirium, coma

## Abstract

Delirium among critically ill patients is common. Presence of delirium imparts a poorer prognosis to patients, including longer ICU and hospital length of stay, increased risk of institutionalization, higher health related costs, and elevated mortality. Even with such grave consequences, the rates of delirium diagnosis are dire. The importance of early recognition through validated tools and appropriate management of this life-threatening condition cannot be over emphasized. This article provides an overview of delirium pathophysiology, diagnosis, and management with a focus on critically ill patients.

## Introduction

Delirium or acute brain failure is the most prevalent psychiatric syndrome found in general hospital setting [1]. In a critical care environment, delirium occurs in up to 60% to 80% of mechanically ventilated medical and surgical intensive care unit (ICU) patients; and in 50% to 70% of non-ventilated patients [2, 3]. The occurrence of delirium in the ICU has prognostic implications, as it has been linked to untoward consequences, like self-extubation, and removal of catheters; greater duration of hospitalizetion; higher mortality; and increased risk of long-term cognitive impairment [4, 11]. Despite these adverse prognostic indicators, delirium has remained largely unrecognized 12].

## Risk Factors

A systematic review of the literature focused on identifying risk factors for ICU delirium found multiple pre-disposing and precipitating risk factors [13]. The predisposing risk factors reported were respiratory disease, age, alcohol abuse and dementia. Twenty one precipitating factors (Table 1) [13, 14] were listed in this report and included disease severity scores, comorbidities, medications (mostly sedatives and opiates) and serum markers. Recently, an association between delirium and drugs with anticholinergic properties was found in a systematic review by Campbell, *et al* [15].

## Pathophysiology

The pathophysiology of delirium is not completely understood. Current evidence suggests that drug toxicity, inflammation and acute stress responses lead to a disruption of neurotransmission, which results in delirium [16]. The final pathway seems to be a combination of increased dopaminergic activity, decreased cholinergic activity, and increased ɣ-aminobutyric acid (GABA)-ergic activity [17]. At present, there are two leading hypotheses explaining delirium development, the neurotransmitter hypothesis and the inflammatory hypothesis [2]. The neurotransmitter hypothesis deals with the deficiency and excess of certain neurotransmitters as mentioned above, and the inflammatory hypothesis emphasizes the role of stress induced cytokines. It is probable that the mechanisms underlying the two hypotheses intersect and feed off each other in producing the final clinical state of delirium [2].

## Clinical Presentation

Delirium is a syndrome of disturbance of consciousness; with deficits in attention; and changes in cognition or perception; that develop over a short period, and fluctuate over the course of the day [18]. Delirium can be classified into distinct subtypes, typically referred to as hypoactive, hyperactive, and mixed [17, 19]. Hypoactive delirium, often unrecognized, is characterized by symptoms of lethargy and minimal psychomotor activity [20]. Hyperactive delirium is marked by significant agitation, restlessness, hypervigilance, and combative behavior. Symptoms of mixed delirium fluctuate between the hypoactive and hyperactive expressions. The most common subtype among ICU patients is mixed (55% of subjects), followed by hypoactive (44%). Less than 2% of critically ill patients have hyperactive delirium [19].

## Management

**Figure 1:**
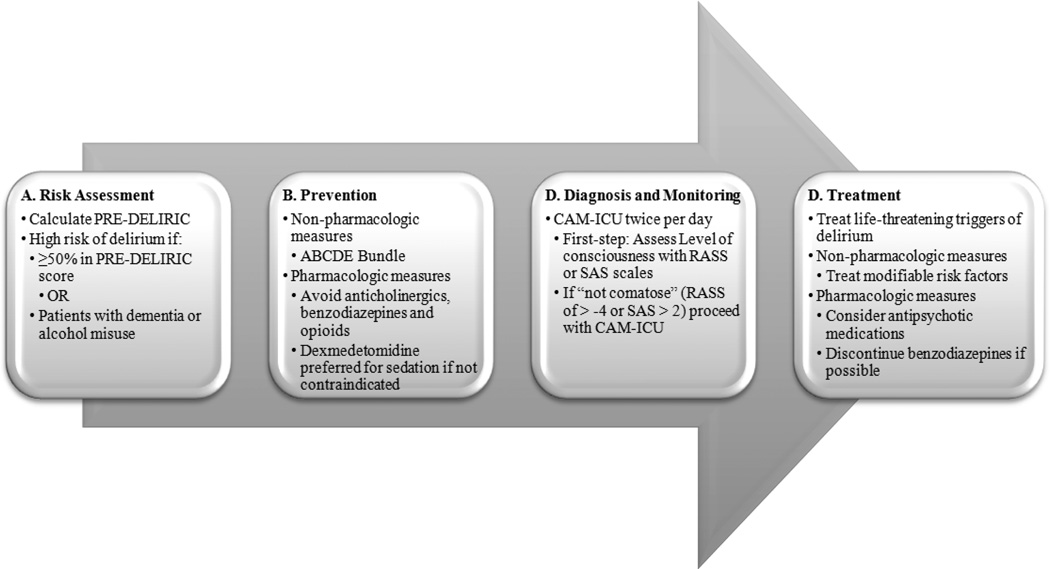
Management of delirium PRE-DELIRIC: PREdiction of DELIRium in ICu patients [21]; ABCDE: Awakening and Breathing Coordination, Delirium Monitoring and Management, and Early Mobility [22, 23]; CAM-ICU: Confusion Assessment Method for the ICU [24, 25]; RASS: Richmond Agitation Sedation Scale [26]; SAS: Riker Sedation-Agitation Scale [27].

A recent systematic evidence review encompassing multiple aspects of delirium has suggested a clinical model for delirium management [2]. This strategy focuses on four main steps: a) risk assessment, b) prevention, c) diagnosis (monitoring), and d) treatment of established delirium (Figure 1).

### Risk Assessment

Majority of patients admitted to the ICU are at high risk, with multiple predisposing and precipitating risk factors (Table 1) [13]. As mentioned in previous publications, most of the ICU patients have at least 10 risk factors for development of delirium [28]. A recent statistical model validated in ICU subjects can reliably predict the development of delirium if applied within the first 24 hours of admission [21]. This tool can be accessed online at: (http://www.umcn.nl/Research/Departments/intensive%20care/Documents/Predeliric%20model.htm?language=english). A perceived importance of early capturing the high-risk patients is the timely institution of preventive measures, with the aim of decreasing the incidence and severity of delirium. This is currently controversial as most of the preventive strategies in the ICU have not been able to prevent delirium, as discussed in the next section.

### Prevention

In practical terms, risk factors for delirium can be divided into three categories: the acute illness itself, host factors including age or chronic health problems, and iatrogenic or environmental factors [28]. Although the first two categories are usually not amenable for modification, the iatrogenic and/or environmental factors can be modified to decrease delirium incidence. A practical step to prevent delirium is to correct the underlying physiologic disturbances as much as possible (e.g. hypoxia, hypotension, electrolyte abnormalities). Non-pharmacologic preventive measures could be implemented to decrease delirium occurrence [29–31]. Few of these measures have been tested in clinical trials in the critical care setting. Noise reduction with earplugs is the only tested intervention, that reduces the incidence of confusion in a critical care population [32]. The remainder of strategies as discussed below has proven to be of benefit in decreasing the incidence, but not the duration of delirium in non ICU populations [29–31].

Non-pharmacologic measures found to be effective in preventing delirium among non-critically ill patients include: repeated reorientation of patients, provision of cognitively stimulating activities multiple times a day, early mobilization, range of motion exercises, timely removal of catheters and physical restraints, use of eye glasses and magnifying lenses, hearing aids and earwax disimpaction, early correction of dehydration, use of a scheduled pain management protocol (not a drip), and minimization of unnecessary noise/stimuli. A blanket recommendation for implementing these measures in the ICU cannot be made based on the current state of literature.

Pharmacologic strategies to prevent delirium should start by avoiding agents that can exacerbate delirium. Randomized trials have demonstrated that delirium related outcomes are improved dramatically when interventions reducing the use of sedatives are employed [33–36]. Specifically, when benzodiazepines are used, the risk of delirium is increased, in contrast to dexmedetomidine which has been associated with less delirium in the ICU [37–41]. Other agents that should be avoided are medications with anticholinergic properties (Table 2). Our group has found an increased association between these pharmaceutical agents and cognitive impairment including delirium in older adults [15, 42]. Other effective strategies have focused on decreasing the amount of sedative use with intermittent boluses v. continuous drips; daily interruption of sedation; sedation protocols targeting specific sedation scores; and analgosedation [33, 43–45]. We believe that these strategies should be implemented in critical care units as standard of care.

Both typical and atypical antipsychotic medications have been tested in multiple studies to prevent delirium [46–49]. Of these studies, only one has been conducted in critically ill patients [49]. In this study of noncardiac surgery patients, a low-dose continuous haloperidol infusion post-surgery was able to reduce the delirium incidence to 15.3% compared to 23.2% in the control group (*P*=0.031). Although the results of this trial seem promising, studies reproducing similar results in a broader patient population in different settings (surgical, medical, trauma) are required before adopting this preventive strategy as standard of care. Currently, we do not advocate the use of prophylactic medications to decrease the incidence of delirium, although this is still a matter of continuous research and debate [48, 50].

Recently, an evidence based strategy combining most of the preventive measures described above, called the ABCDE (Awakening and Breathing Coordination, Delirium Monitoring and Management, and Early Mobility) bundle has been employed successfully at multiple institutions to decrease the burden of delirium [22,23]. Previous studies have demonstrated the positive impact of performing daily spontaneous awakening trials (SAT) –A in the bundle-by facilitating the transition from drug-induced coma to consciousness, reducing the duration of mechanical ventilation, and reducing ICU complications and costs [33, 34, 51]. Spontaneous breathing protocols (SBT), when safe and indicated, have shown to decrease ventilator days and complications derived from mechanical ventilation (B in the bundle) [52, 53]. Coordinating daily SATs with daily SBTs has been associated with decreased adverse cognitive outcomes, reduced hospital length of stay and reduced 1-year mortality (C in the bundle) [35]. Delirium monitoring (D portion of the bundle), leading to improved recognition and institution of management strategies as mentioned in the following sections. Last, early mobilization (E of the bundle) of ICU subjects has shown to decrease the duration of delirium [54]. The feasibility of mobilization in the critical care setting has been demonstrated in multiple reports [55, 56].

### Diagnosis

Once patients at risk for delirium are identified, and their modifiable risk factors mitigated, the next step in the management of ICU delirium is to monitor frequently for its occurrence (D from the ABCDE bundle) [57]. The gold standard for diagnosing delirium is through clinical history and focused examination, as guided by the Diagnostic and Statistical Manual of Mental Disorders-Fourth Edition (DSM-IV). Based on the DSM-IV definition, the criteria required for the diagnosis are cognitive change, and disturbance in consciousness, which should develop in a short period of time. However, the nature of underlying illnesses and lack of verbal communication in ICU patients make delirium assessment in the ICU, using DSM-IV criteria, particularly difficult. Fortunately there are well validated tools for the screening and diagnosis of delirium specially designed for ICU subjects. The two most commonly used tools are the Intensive Care Delirium Screening Checklist (ICDSC) [58] and the Confusion Assessment Method for the ICU (CAM-ICU) [25]. The ICDSC is a highly sensitive tool (99%), but with a low specificity (64%). It has a total score ranging from 0 to 8, with delirium defined as a score of 4 or more. The problem with this scale is its high false-positive rate; limiting its use as a screening rather than a diagnostic tool. We prefer the use of the CAM-ICU for the diagnosis of delirium, because of its strong psychometric properties [high sensitivity (93–100%), specificity (89–100%), and inter-rater reliability (k = 0.96,)] [24, 25]. Assessing a patient’s delirium utilizing the CAM-ICU is a two-step process; the first step is to assess the patient’s level of consciousness. The purpose of this step is to identify patients that are in a deeper level of consciousness (coma or stupor), because those subjects would not be eligible to be assessed with the CAM-ICU. The tool that has been used typically to assess consciousness is the Richmond Agitation Sedation Scale (RASS), a 10-point scale ranging from −5 (no response to voice or physical evaluation) to +4 (overtly combative, violent, immediate danger for staff) (Table 3) [26]. Patients with RASS scores of −4 or −5 are deemed as comatose, and hence, are not eligible to be assessed by the CAM-ICU. Although the RASS score has been used widely with the CAM-ICU, a recent report validated the use of the Riker Sedation-Agitation Scale (SAS) [27] (Table 4) with the CAM-ICU [59]. Both RASS and SAS showed remarkable similarity in identifying patients eligible for CAM-ICU assessment with a rank correlation coefficient of 0.91 and the kappa agreement of 0.93. Based on this report, both scales can be used interchangeably alongside the CAM-ICU for the diagnosis of delirium [59]. Depending upon the scale, patients are considered comatose if RASS is ≤ − 4 or if SAS is ≤ 2, and thus unable to be assessed by CAM-ICU.

**Figure 2:**
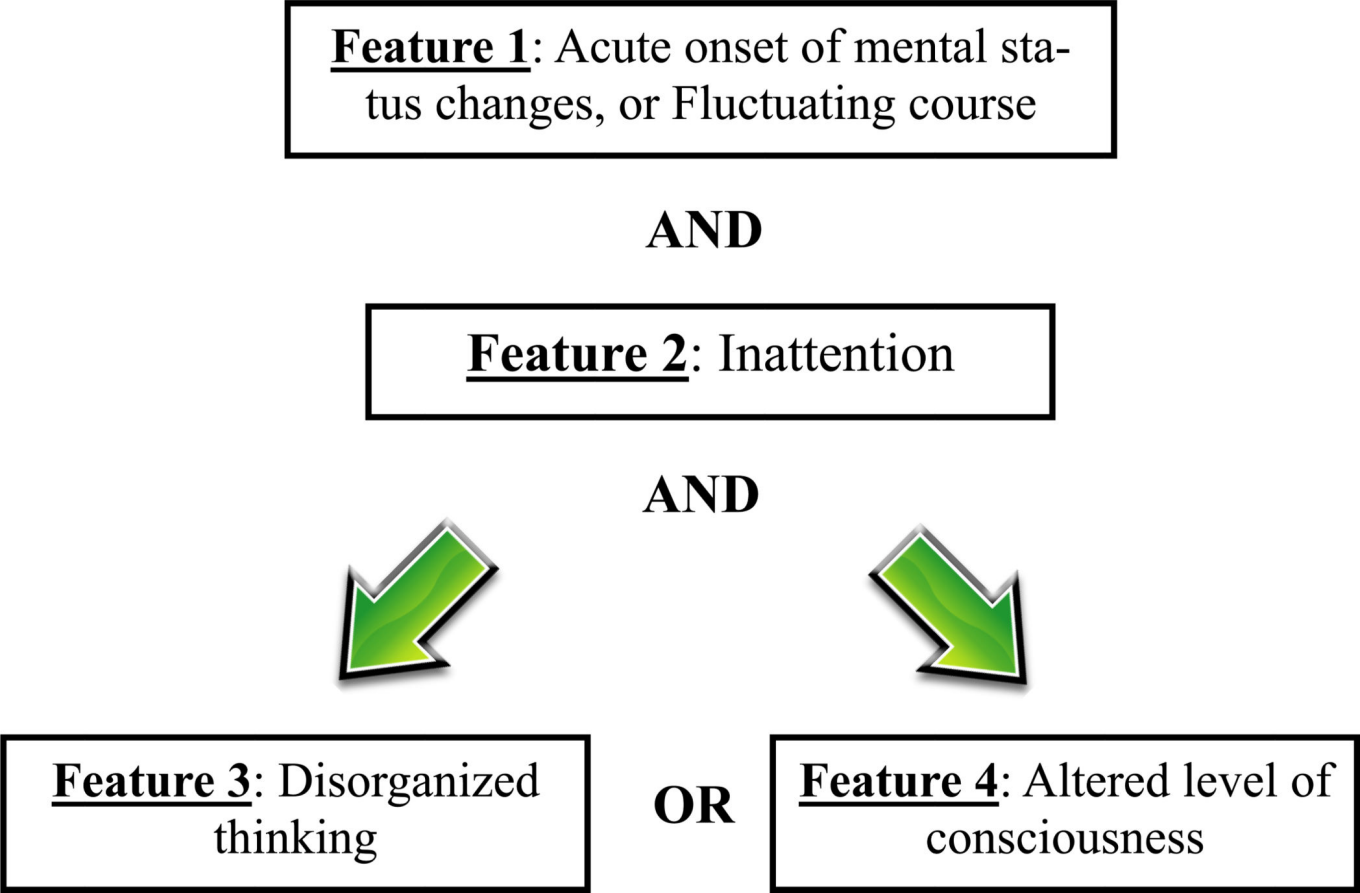
CAM-ICU

Once the patient is deemed “not comatose” or at higher levels of consciousness (RASS of > −4 or SAS > 2), step two of delirium assessment using the CAM-ICU is performed. CAM-ICU consists of four features; an acute change or fluctuation in mental status (**Feature 1**), accompanied by inattention (**Feature 2**), and either disorganized thinking (**Feature 3**) OR altered level of consciousness (**Feature 4**, Figure 2) [24, 25]. A more detailed description of the CAM-ICU and training materials to implement it can be found on the website www.ICUdelirium.org. The optimal frequency of delirium monitoring is still not established, but given the fluctuant nature of the condition, it is advisable to screen for delirium at least twice per day.

### Treatment

Delirium treatment is usually divided into non-pharmacologic and pharmacologic therapies. Most of the non-pharmacologic strategies include efforts to decrease or treat the modifiable risk factors as a first step in management, after delirium has been diagnosed (e.g. decreasing or stopping sedation; optimal pain control; avoiding excessive use of benzodiazepines and opiates). It is of utmost importance to recognize that delirium could be a manifestation of an acute, life-threatening problem that requires immediate attention (hypoxia, hypercarbia, hypoglycemia, metabolic derangements, or shock).

Pharmacologic therapies for delirium are limited. Even though there are no FDA approved medications for the treatment of delirium, antipsychotic agents are widely used in the ICU for the treatment of this syndrome [60]. This use of antipsychotics is based on theoretical reasoning, as there have been no multicenter randomized controlled trials in critically ill patients showing improved outcomes in delirious subjects treated with antipsychotics. Because it is thought that the pathophysiology of delirium is based on neurotransmitter imbalances, antipsychotics may help by restoring the balance. Haloperidol (a “typical” antipsychotic) is widely used and is recommended by Society of Critical Care Medicine guidelines as the drug of choice for treatment of delirium in critically ill patients [57]. It acts by blocking the dopamine D2 receptors, thereby decreasing the dopaminergic load. In contrast, the “atypical” antipsychotic agents (e.g. aripiprazole, olanzapine, quetiapine, and ziprasidone) affect several different neurotransmitters, including norepinephrine, serotonin, histamine, and acetylcholine, giving them a theoretical advantage over haloperidol. In practice, atypical antipsychotics have not shown to be superior in efficacy for delirium treatment, with studies attesting to the equal efficacy of atypical antipsychotics and haloperidol [61]. Skrobik, *et al*. reported that both olanzapine and haloperidol had the same effectiveness in reducing delirium (although there was no placebo arm in the study), but haloperidol was associated with more extrapyramidal symptoms [62]. Use of cholinesterase inhibitors in the ICU has been associated with increased mortality based on a trial that was stopped early due to increased mortality in the rivastigmine arm compared to placebo [63]. Table 5 provides the recommended dosing for antipsychotics based on current literature.

There are two major randomized, placebo-controlled trials of antipsychotics for treatment of delirium in ICU subjects. The MIND (Modifying the Incidence of Delirium) trial randomized 101 patients to receive haloperidol, ziprasidone, or placebo every 6 hours for up to 14 days. It concluded that there were no differences between groups in the duration of delirium or days alive without delirium [65]. None of the antipsychotics were superior compared to placebo in treating delirium. The second study looked at 36 ICU patients with delirium who were assigned to receive either quetiapine or placebo in addition to as needed haloperidol. Quetiapine was associated with a shorter “time to first resolution” and reduced duration of delirium. In addition, the quetiapine group required less rescue haloperidol than the placebo group [64]. A major limitation of both studies was their small sample size. Currently there are two trials in critically ill delirious patients testing antipsychotics [66, 67]. One of them, the PMD (Pharmacological Management of Delirium) is a pragmatic, effectiveness trial, testing a multicomponent intervention (reduction in benzodiazepine and anticholinergic medication use, plus low dose haloperidol) in decreasing delirium duration and severity versus usual care. The other, MIND-USA (Modifying the Impact of ICU-Associated Neurological Dysfunction) is a three arm efficacy trial comparing haloperidol, ziprasidone, and placebo in improving delirium/coma free days. The results of these trials will provide further direction regarding the use of these agents in critically ill patients [66, 67].

## Conclusion

Delirium is a common syndrome in critically ill subjects characterized by impairment in cognition and behavior. Delirium is often multifactorial and is related to underlying medical, environmental and iatrogenic factors. The diagnosis of delirium is challenging with frequent under recognition. CAM-ICU is the best tool for diagnosis of delirium in the ICU. Untreated, delirium can have devastating consequences and its occurrence is associated with increased morbidity and mortality. Available evidence indicates that early detection, reduction of risk factors, and better management of this condition can decrease its morbidity rates.

## Figures and Tables

**Table 1:** †Risk Factors for Delirium in Critical Care

Host Factors	Factors of Critical Illness	Iatrogenic Factors
Age (Higher age means higher risk)	Anemia	Coma (with the use of medication)
Alcohol abuse	*APACHE II score (Higher score has increased risk)	Dopamine administration
Anemia	Azotemia	Epidural catheter use
Dementia / Cognitive impairment	Elevated hepatic enzymes	Lorazepam (or other benzodiazepines)
Visual/Hearing impairment	Fever	Morphine (Or other opiates)
	Hyperamylasemia	
	Hyperbilirubinemia	
	Hypertension	
	Hypocalcaemia	
	Hyponatremia	
	Hypotension	
	Infections	
	Metabolic acidosis	
	Respiratory disease	

† The risk factors are incorporated from references [13] and [14].

* APACHE: Acute Physiology and Chronic Health Evaluation.

**Table 2:** Anticholinergic cognitive burden scoring of drugs

Score 1	Score 2	Score 3
Alimemazine	Amantadine	Amitriptyline
Alverine	Belladone alkaloids	Amoxapine
Alprazolam	Carbamazepine	Atropine
Atenolol	Cyclobenzaprine	Benztropine
Brompheniramine maleate	Cyproheptadine	Brompheniramine
Bupropion hydrochloride	Empracet	Carbinoxamine
Captopril	Loxapine	Chlorpheniramine
Chlorthalidone	Meperidine	Chlorpromazine
Cimetidine hydrochloride	Methotrimeprazine	Clemastine
Ranitidine	Molindone	Clomipramine
Clorazepate	Oxcarbazepine	Clozapine
Codeine	Pethidine hydrochloride	Darifenacin
Colchicine	Pimozide	Desipramine
Coumadin		Dicyclomine
Diazepam		Dimenhydrinate
Digoxin		Diphenhydramine
Dipyridamole		Doxepin
Disopyramide phosphate		Flavoxate
Fentanyl		Hydroxyzine
Furosemide		Hyoscyamine
Fluvoxamine		Imipramine
Haloperidol		Meclizine
Hydralazine		Nortriptyline
Hydrocortisone		Olanzapine
Isosorbide		Orphenadrine
Loperamide		Oxybutynin
Metoprolol		Paroxetine
Morphine		Perphenazine
Nifedipine		Procyclidine
Prednisone		Promazine
Quinidine		Promethazine
Risperidone		Propentheline
Theophylline		Pyrilamine
Trazodone		Quetiapine
Triamterene		Scopolamine
		Thioridazine
		Tolterodine
		Trifluoperazine
		Trihexyphenidy

Drugs with possible anticholinergic effects have a score of 1. Drugs with established and clinically relevant cognitive anticholinergic effects have either a score of 2 or 3, based on the drug blood-brain barrier permeability and its association with the development of delirium. Drugs with no anticholinergic effects can be considered as having a score of zero. The total added score of different drugs taken by the patient determines the cumulative Anticholinergic Cognitive Burden. A total Anticholinergic Cognitive Burden scale score of ≥ 3 is considered clinically relevant [42].

**Table 3:** Richmond Agitation-Sedation Scale

Score	Term	Description
+4	Combative	Overtly combative, violent, danger to staff
+3	Very agitated	Pulls or removes tubes or catheters; aggressive
+2	Agitated	Frequent nonpurposeful movement, fights ventilator
+1	Restless	Anxious, but movements not aggressive or vigorous
0	Alert and calm	…
−1	Drowsy	Not fully alert, but has sustained awakening (eye opening/eye contact) to voice (> 10 s)
−2	Light sedation	Briefly awakens with eye contact to voice (< 10 s)
−3	Moderate sedation	Movement or eye opening to voice (but no eye contact)
−4	Deep sedation	No response to voice, but movement or eye opening to physical stimulation
−5	Unable to rouse	No response to voice or physical stimulus

**Table 4:** Sedation-Agitation Scale

Score	Term	Description
7	Dangerous agitation	Pulling at endotracheal tube, trying to remove catheters, climbing over bed rail, striking at staff, thrashing side to side
6	Very agitated	Does not calm, despite frequent verbal reminding of limits; requires physical restraints, biting endotracheal tube
5	Agitated	Anxious or mildly agitated, attempting to sit up, calms down to verbal instructions
4	Calm and coopera tive	Calm, awakens easily, follows commands
3	Sedated	Difficult to arouse; awakens to verbal stimuli or gentle shaking, but drifts off again; fol lows simple commands
2	Very sedated	Arouses to physical stimuli, but does not communicate or follow commands, may move spontaneously
1	Unable to rouse	Minimal or no response to noxious stimuli, does not communicate or follow commands

**Table 5:** Antipsychotic medications dosing (based on previous reports)

Medication	Suggested Dose
Haloperidol	2–5 mg (0.5–2 mg in the elderly) intravenously, followed by double repeated doses every 15–20 min if agitation persists up to a maximum of 20 mg/d [57]
Olanzapine	Starting dose 5 mg (2.5 mg over 65 years) and titrated on clinical judgment [62]
Risperidone	Starting dose 0.5 mg twice a day, up to a maximum of 2.5 mg/d [64]
